# Interval Fuzzy Model for Robust Aircraft IMU Sensors Fault Detection

**DOI:** 10.3390/s18082488

**Published:** 2018-08-01

**Authors:** Michele Crispoltoni, Mario Luca Fravolini, Fabio Balzano, Stephane D’Urso, Marcello Rosario Napolitano

**Affiliations:** 1 Dipartimento di Ingegneria, Università di Perugia, Via G. Duranti, 67, 06125 Perugia, Italy; michele.crispoltoni@studenti.unipg.it (M.C.); fabio.balzano@studenti.unipg.it (F.B.); 2 Department of Mechanical and Aerospace Engineering, West Virginia University, Morgantown, WV 26506-6106, USA; stdurso@mix.wvu.edu (S.D.); marcello.napolitano@mail.wvu.edu (M.R.N.)

**Keywords:** fault detection, fuzzy interval models, linear matrix inequalities, inertial navigation system, data-driven modelling

## Abstract

This paper proposes a data-based approach for a robust fault detection (FD) of the inertial measurement unit (IMU) sensors of an aircraft. Fuzzy interval models (FIMs) have been introduced for coping with the significant modeling uncertainties caused by poorly modeled aerodynamics. The proposed FIMs are used to compute robust prediction intervals for the measurements provided by the IMU sensors. Specifically, a nonlinear neural network (NN) model is used as central prediction of the sensor response while the uncertainty around the central estimation is captured by the FIM model. The uncertainty has been also modelled using a conventional linear Interval Model (IM) approach; this allows a quantitative evaluation of the benefits provided by the FIM approach. The identification of the IMs and of the FIMs was formalized as a linear matrix inequality (LMI) optimization problem using as cost function the (mean) amplitude of the prediction interval and as optimization variables the parameters defining the amplitudes of the intervals of the IMs and FIMs. Based on the identified models, FD validation tests have been successfully conducted using actual flight data of a P92 Tecnam aircraft by artificially injecting additive fault signals on the fault free IMU readings.

## 1. Introduction

The occurrence of failures on the different classes of sensors in a flight control system is a very critical issue from a safety and reliability point of view. Particularly critical is the fault detection (FD) of sensors whose measurements are used in real time in the feedback of the aircraft flight control laws. This paper presents a data-driven approach for the fault detection of inertial measurement unit (IMU) sensors, in particular for the angular rates sensors. An IMU is the main component of the inertial navigation system (INS) and is used by the flight computer to calculate attitude, angular rates, linear velocity, and position with respect to a global reference frame. IMUs are used on both manned and unmanned aircraft for navigational purposes in particular during poor weather conditions as well as in the event of lack of ground communication. The navigation errors of an INS are mainly caused by issues such as the gyroscope drifts and accelerometer bias. Because the guidance system is continually integrating acceleration with respect to time to calculate airspeed and position (dead reckoning), measurement errors, even if small, accumulate substantially over time. This leads to the well-known drifting error, which is essentially an ever-increasing difference between the system “perceived” and actual location. Thus, a constant error in attitude rate (gyro) eventually leads to a quadratic error in airspeed and a cubic error growth in position. To deal with these issues sensor fusion techniques with other navigation systems such as global positioning systems (GPS) and magnetometers are commonly implemented to provide an accurate and reliable state estimation [1]. However, there might be flight zones and/or specific situations with malfunctions of the above instruments when the IMU operates as a stand-alone. Thus, ensuring fault-free and stable operation of this sensor system is crucial for safe and reliable tracking to a designated flight trajectory.

According to the final reports of crash investigations a significant number of flight accidents has been attributed to INS failures, such as the crashes of the Qantas F72, Croatia Boeing 737-200, and Adam Air 574, just to cite a few [2]. Specifically, the final report released by the Indonesian National Transport Safety Committee specifically mentions that Adam Air Flight 574 crashed because the pilots were confused by the readings in the cockpit following the failure of the Boeing 737 inertial reference system (IRS).

The main purpose of this paper is to propose the design of an FD system providing a fast fault detection of IMU sensors along with desirable robustness to false alarms. In general, the reliability of aircraft instrumentation mandated by the regulating agency is typically achieved through hardware physical redundancy (HPR) [3]. HPR consists in the installation of multiple sensors (originally 4, later reduced to 3) with a voting logic used to detect, identify, isolate, and exclude the faulty sensor. Clearly, HPR implies an increase in the complexity of the on-board instrumentation whose cost and weight may be acceptable only for commercial aviation. In case of small aircraft and/or unmanned aerial vehicles (UAVs) where weight, power consumption, dimensions, costs, and overall system complexity are key design requirements an alternative solution is provided by FD schemes based on analytical redundancy (AR) [4]. In general, AR-based redundancy is achieved through the use of a mathematical model that provides, online, a fault free estimation of the possibly faulty measurement. Additionally, the output of this model is used to derive a diagnostic signal (residual) as the difference between the measurement and the estimation provided by the model. A recent application of AR techniques to the FD of actuators and sensors on UAVs can be found in [5].

Clearly, the performance of the FD system is strictly related to the level of model uncertainty that is quantified by the residual signal. There are many factors that influence this unavoidable uncertainty, such as variability in physical parameters, unmodeled and/or poorly modeled dynamics, time delays, nonlinearities (of either dynamic or aerodynamic nature), noise, drifts and offsets in the sensors, as well as external disturbances. The consequence of the modeling uncertainty is that even in fault free conditions the residual signal deviates from zero; therefore, the development of accurate estimation models and the characterization of the uncertainty bounds are equally important.

The concept of a bounded error estimation has attracted substantial interest in the FD and diagnostics research community leading to the development of the so-called set membership or interval model (IM) methods [6,7,8].

Within IM approaches, the uncertainty in the model parameters is translated into time-varying bounds (thresholds) for the estimation that are computed by varying the uncertain parameters within defined bounded intervals. The presence of potential faults or anomalies is detected on-line when the measured signal falls outside the time varying upper and lower bounds. The IM general concept has also been investigated in the area of fuzzy modeling where upper and lower membership functions along with weighting coefficients are used to characterize the uncertainties in the so called interval type-2 fuzzy approach [9,10].

An important contribution of this paper is in the development of a practical procedure for IMs identification formulated as a convex optimization problem whose decision variables are the amplitudes of the intervals and whose cost function is the mean amplitude of the prediction intervals. Specifically, the optimization is formalized as a linear matrix inequality (LMI) problem that can be efficiently solved using a conventional convex optimization software. The procedure has been here used to identify both conventional fixed amplitude Internal models (IMs) and more flexible models allowing the uncertainty amplitude to depend on one or more variables (in this case the aircraft altitude). This flexibility was achieved through a fuzzy fusion of a fixed number of local linear IMs models of the uncertainty as function of the aircraft altitude. The resulting model is here referred to as fuzzy interval model (FIM).

The proposed identification procedures for the IMs and FIMs are both a generalization of the IM identification procedure proposed in [11] where the optimization of the uncertainty box is based on a scalar free parameter *λ* used to scale a predefined shape uncertainty box domain. The approach in [11] is less complex but has the drawback of producing likely a conservative box because the same numerical value of *λ* is used to scale the size of the interval box along all the directions in the same way. Instead, IM and FIM methods are designed to scale the dimensions of the uncertainty box independently and, therefore, the net effect is that they are able to produce much tighter uncertainty domains.

The IM and FIM identification procedure was validated using real multiple flight data of a P92 Tecnam aircraft [12]. Quantitative tests have shown that FIMs are particularly robust in capturing the actual nonlinear response of the aircraft, with bounding prediction intervals tighter than those provided by conventional IMs. This fact is very relevant from a FD point of view since tighter intervals allow the detection of smaller amplitude faults.

Additionally, to highlight the effectiveness of FD schemes based on IMs and FIMs, a side-by-side performance comparison was conducted with conventional FD approaches based on fixed detection thresholds [13] that have been inferred from the statistical analysis of the fault free modeling error (residual signal) produced by conventional deterministic identification models.

In summary, the innovative aspects of this study are, first, to show that the proposed data-driven IM and FIM identification technique can be applied with success to real life problems to compute robust time varying FD thresholds for sensors and, second, that the performance of FD schemes based on FIMs and IMs are significantly better than those provided by conventional (single value) models based on fixed thresholds.

The paper is organized as follows. Section 2 introduces the nonlinear and linear regression models used to characterize the nominal response of the IMU sensors. Section 3 describes the IMs, the FIMs, and the data-driven optimized parameters identification procedure. Section 4 provides a brief overview of the P92 aircraft and its flight data, while Section 5 describes the procedure for selecting the modeling regressors. Section 6 reports the central models identification results. Section 7 summarizes the IM and FIM identification results. Section 8 discusses failure modeling, while Section 9 illustrates the validation results of the proposed FD architectures using experimental data from 4 different flights. Final conclusions are drawn in Section 10.

## 2. Experimental Identification Models for the IMU Sensors

The conventional approach for deriving experimental models for predicting the measurement of sensors as function of correlated signals requires first the selection of a suitable linear or nonlinear in the parameters model structure; this selection is followed by the identification of the model parameters through a specific algorithm using a batch of representative “rich” training data. In a fault detection context, the identified prediction model, at times referred to as “virtual sensor”, is then used on-line to compute a diagnostic signal (known as residual) that is the difference between the actual measurement and its estimation, whose abnormal deviation could be attributed to a potential fault. Clearly, the more accurate and reliable a model is, the simpler will be the detection of small amplitude faults while maintaining a low level of false alarms. The first identification model proposed in this paper is based on conventional nonlinear regression models identified from input–output data. These models, also named as “central” models, provide an estimation of the measured signal; however, they do not provide any information about the modeling uncertainty and its effects on the prediction. Specifically, the following classes of “conventional models” have been considered.

### 2.1. Nonlinear Regression (NR) Model

It is assumed that an output variable *y*(*k*), which in our case is the potential faulty IMU signal, can be estimated as function of *n* different measurable signals *x*(*k*) = [*x*_1_(*k*), *…*, *x_n_*(*k*)] correlated with the *y*(*k*) dynamics. The most general structure used in this study is a generic input-output nonlinear regression (NR) model of the form [14,15]:

(1) $$y(k)=\hat{y}(k)+\varepsilon (k)=f_{nn}(\varphi (x(k),W_{nn})+\varepsilon (k)\;$$

where $\hat{y}(k)$ is the estimation of *y*(*k*) at sample time *k* and *ϕ*(*k*) is a *p* × 1 vector of linear and nonlinear functions of *x*(*k*) and of its delayed values *x*(*k* − *j*), *j* = 1, *…*, *m* (where *j* represents the number of delay steps); *ε*(*k*) represents the modeling error. The *f_nn_*(∙) function is a generic nonlinear parametric universal approximation structure; in this paper it has been selected to be a Neural Network (NN) approximator [16]. Finally, *W_nn_* is the vector of the NN parameters to be computed in the model identification phase. It should be pointed out that Equation (1) does not depend on *y*(*k* − *j*). Therefore, the estimation $\hat{y}(k)$ is not affected by the occurrence of a fault on *y*(*k*), thus avoiding the “fault following” effect [11]. In other words, the fault itself can affect the estimation which, in turn, influences the residual ($r(k)=y(k)-\hat{y}(k)$) with the net effect that it quickly adapts itself to the faulty conditions resulting, therefore, insensitive to the fault even if the fault is persistent. Clearly, this effect is undesirable. Hence, the NR in Equation (1) is able to provide a reliable long term estimation independently of the occurrence of a sensor fault affecting *y*(*k*).

In case the model (1) is linear in the functions *ϕ*(*k*), the above relationship reduces itself to the following well-known linear regression model [17]:

(2) $$y(k)=\hat{y}(k)+\varepsilon (k)=\varphi^{T}(k)W_{LR}+\varepsilon (k)\;$$

where the vector $W_{LR}\in p\times 1$ collects the *p* parameters of the linear model.

NOTE: The adoption of the nonlinear model (1) or the linear model (2) cannot be established ‘a-priori’ but, rather, is the result of an analysis that, in general, depends on the specific problem. Even if the simpler linear models are preferable due to the simplicity in the identification process along with a lower computational effort required for real-time implementation, in the present study the Nonlinear Regression models have been preferred because the NNs models showed to be able to better approximate the experimental responses of the IMU sensors throughout the entire flight envelope of the aircraft when compared to the simpler linear models.

### 2.2. Regressors Selection and Conventional Models Identification

The selection of the set of modeling regressors in *ϕ*(*k*) to be used in Equations (1) and (2) is based on the assumption that the identifying functions admit a polynomial approximation as function of the of signals *x*(*k*) = [*x*_1_(*k*), *…*, *x_n_*(*k*)]. In other words, it is assumed that *ϕ*(*k*) contains a collection of monomial terms of the form $x_{1}^{\alpha_{1}}(t)\cdot x_{2}^{\alpha_{2}}(t),\ldots ,\cdot x_{n}^{\alpha_{n}}(t)$ where the coefficients $\alpha_{i},i=1,\ldots ,n$ are integers exponent (including zero) to be defined by the model identification process. Clearly, the selection of the modeling regressors is a critical issue for deriving reliable models. This step is often performed using specific algorithms that iteratively select and include in the model an increasing number of regressors until an ‘a priori’ defined level of accuracy for the modelling prediction error is achieved for a given set of training data. In this study the “stepwise selection algorithm” has been used [18]. Following the regressors selection phase, the identification of the models (1) was performed using multilayer feedforward NN models trained with a supervised learning algorithm using experimental multiflight data along with the tools of the MATLABNN toolbox [19].

## 3. Interval Models

This section describes the IMs and FIMs and the optimized approach used to identify their parameters. The proposed architectures feature as central model the nonlinear regression (NR) models of the form (1) plus an IM or FIM contribution to capture the modeling uncertainty. Specifically, the modeling uncertainty (essentially the error between the actual *y*(*k*) output and the predicted output $\hat{y}(k)$) is modeled as a bounded contribution that, in turn, depends linearly on the regresses vector set *ϕ*(*k*) through a set of uncertain, but bounded, parameters that capture the uncertainty effects.

### 3.1. The Conventional Interval Model (IM)

The conventional IM estimator uses as central model a Neural Network approximation *f_nn_*(∙) of the form (1) plus a linear in the parameter IM to capture the residual modeling uncertainty around the central model. The resulting model is of the form $\hat{y}(k)=f_{nn}(\varphi (k),W_{nn})+∆y(k)$**,** where $∆y(k)=\;\varphi^{T}(k)∆w$ is an uncertainty term linearly parametrized as function of a vector ∆w of uncertain but bounded parameters $∆w_{i}$ that belong to a bounded set $∆\bar{W}$. Since the parameters $∆w_{i}$ are uncertain, the estimation provided by the model $\hat{y}(k)$ is not a specific numerical value but, rather, an interval of values. In the particular case the parameters $∆w_{i}$ belong to a rectangular box set, then the $∆\bar{W}$ set is defined by the intervals

(3) $$∆w_{i}\in [-∆{\bar{w}}_{i},+∆{\bar{w}}_{i}]i=1,\ldots ,p\;$$

where the $∆{\bar{w}}_{i}$ are the maximum amplitudes of the of the parametric uncertainty associated with the *i*-th regressor. The estimation $\hat{y}(k)$ is bounded within the following upper and lower bounds:

(4) $$\begin{array}{l} \bar{y}(k)=f_{nn}(\varphi (k),W_{nn})+|\varphi^{T}(k)|∆W\;∆W\in ∆\bar{W}\\ \underline{y}(k)=f_{nn}(\varphi (k),W_{nn})-|\varphi^{T}(k)|∆W\;∆W\in ∆\bar{W} \end{array}\;$$

where ∆W is a vector containing the $∆{\bar{w}}_{i}$ values. In this study, the amplitudes $∆{\bar{w}}_{i}$ are ‘a priori’ unknown and will be identified from data following the optimized procedure described in the next section.

### 3.2. Identification of the Conventional IM

The computation of the unknown parameters $\left[∆{\bar{w}}_{1},∆{\bar{w}}_{2},\ldots ,∆{\bar{w}}_{p}\right]$ that characterize the uncertainty associated with the IMs plays a central role in this study. The identification of these parameters should meet the goal of providing a reliable and, at the same time, non-conservative estimation of the uncertainty. These objectives were both achieved following the design principles proposed in [20], that is the *consistency* with the set of all the training data and the interval *tightness* criterion. Considering the consistency requirement, the output *y*(*k*) of the IM model, must satisfy the condition:

(5) $$\bar{y}(k)\leq y(k)\leq \underline{y}(k)k=1,\ldots ,N\;$$

for each sample for *k* = 1, …, *N*. As a result, the IM prediction interval (5) is able to capture the uncertainty affecting the model in the sense that all the training data are consistent with the bounds produced by the IM without any sample outside the prediction interval. The *tightness* requirement implies that the IM identification procedure has to provide an uncertainty set $\left[∆{\bar{w}}_{1},∆{\bar{w}}_{2},\ldots ,∆{\bar{w}}_{p}\right]$ as tight as possible around the central model while being consistent with all the training data according to the consistency criterion (5). The tightness requirement has been formalized through the introduction of a cost function $J=E(\bar{y}(k)-\underline{y}(k))$, which is essentially the mean of the output prediction interval produced by the training data.

By combining the consistency and the tightness requirements the identification of the parameters $∆W_{i},i=1,\ldots ,p$ has been converted into a constrained minimization problem of the *J* index. Specifically, the goal of the optimization is to compute the minimum mean amplitude prediction interval *J* that is consistent with all the *N* input–output training data. This has been rigorously formalized as the following linear matrix inequality (LMI) minimization:

(6) $$\begin{array}{l} \underset{∆{\bar{w}}_{1},∆{\bar{w}}_{2},\;\ldots ,\;∆{\bar{w}}_{p}}{\min}J\;subject\;to\\ \;+y(k)-f_{nn}(\varphi (k),W_{nn})-|\varphi^{T}(k)|∆W<0\;\;\;k=1,\;\ldots ,\;N\\ \;-y(k)+f_{nn}(\varphi (k),W_{nn})-|\varphi^{T}(k)|∆W<0 \end{array}\;$$

The optimization problem in (6) is convex in the unknown parameters $∆W_{i},i=1,\ldots ,p$ and can be efficiently solved with a number of optimization codes. The solution of the optimization problem provides the minimum uncertainty box $∆{\bar{w}}_{i},i=1,\ldots ,p$ satisfying the consistency requirement. It should be emphasized that the LMI problem (6) is always solvable; in fact, it is always possible to compute “large enough” values for the unconstrained parameters $∆{\bar{w}}_{i},i=1,\ldots ,p$ that are consistent with all the training data.

### 3.3. The Fuzzy Interval Model (FIM)

In the conventional IM approach (3) the uncertainty amplitudes $∆{\bar{w}}_{i}$ are constant parameters that are independent of the specific operating conditions of the system. On the other hand, it could be more effective to have a more flexible model where the amplitudes of the uncertainty may depend on the specific operating conditions. For instance, for the purposes of our study, it could be argued that the amplitudes of the $∆{\bar{w}}_{i}$ parameters are likely to be different at take-off, level flight, and descent/approach/landing conditions. Clearly, this flexibility may allow us to achieve tighter prediction intervals with obvious improvements in terms of modelling accuracy. This flexibility has been achieved through the introduction of an approach where *m* local IMs of the form (3) are fused together using a fuzzy fusion strategy. The considered Interval Fuzzy Model (IFM) was originally introduced in [21]. Specifically, the overall fuzzy model for the uncertainty is based on *m* local models $∆y_{i}(k)$ that are defined by a set of *m* fuzzy rules $R_{j}$ defined as follows:

(7) $$R_{j}:\;\mathrm{if}x_{p}(k)\mathrm{is}\;A_{j}\mathrm{then}\;∆y_{j}\left(k\right)\;=\;\varphi {\left(k\right)}^{T}∆W_{j}\beta_{j}\left(x_{p}(k)\right)\;j\;=\;1,\ldots ,m$$

where the variable *x_p_*(*k*) denotes the antecedent (or premise) input variables, *ϕ*(*k*) is the vector of *p* regressors, $∆W_{j}$ (*j* = 1, …, *m*) are modelling parameter vectors defining the *j* fuzzy model, and $A_{j}$ (*j* = 1, …, *m*) are ‘a-priori’ defined fuzzy sets characterized by real-valued membership functions $\mu_{A_{j}}(x_{p}):\;R\to \left[0,1\right]$ providing the activation of the *j*-th fuzzy rule evaluated at the working point $x_{p}(k)$. Finally, $\beta (x_{p})=\left[\beta_{1}(x_{p}),\ldots ,\beta_{m}(x_{p})\right]$ is a vector of normalized activations defined as:

(8) $$\beta_{j}(x_{p})=\frac{\mu_{A_{j}}(x_{p})}{\sum_{j=1}^{m}\mu_{A_{j}}(x_{p})}\;$$

The (output) $∆y_{fuzzy}(k)$ of the overall fuzzy model for the uncertainty is then computed as the weighed combination of *m* local linear models as follows:

(9) $$∆y_{fuzzy}\left(k\right)\;=\;\sum_{j=1}^{m}\left[\varphi^{T}(k)∆W_{j}\right]\beta_{j}\left(x_{p}(k)\right)\;$$

Extending the definition of upper and lower bounds used for the IM in (3), it is immediate to derive the corresponding upper and lower bounds for the FIM as follows:

(10) $$\begin{array}{l} \bar{y}(k)=f_{nn}(\varphi (k),W_{nn})+\sum_{j=1}^{m}\left[|\varphi^{T}(k)|∆W_{j}\right]\beta_{j}\left(x_{p}(k)\right)\\ \underline{y}(k)=f_{nn}(\varphi (k),W_{nn})-\sum_{j=1}^{m}\left[|\varphi^{T}(k)|∆W_{j}\right]\beta_{j}\left(x_{p}(k)\right)\;\\ \; \end{array}\;$$

### 3.4. Identification of the Fuzzy Interval Model

As for the FIMs, in this effort we assumed that the set of the *m* fuzzy membership functions $\mu_{A_{j}}(x_{p})$ is defined ‘a priori’ at design stage (for instance we introduced a set of *m* = 6 Gaussian membership functions defined in Section 7.2, while data-driven identification tools were used to compute optimized values for the parameters that characterize the *m* local models of the uncertainty. Specifically, the identification of the FIM model in (9) requires the computation of the components of the *m* vectors $∆W_{j}$ associated with the *m* local linear models $∆y_{i}(k)$ of the uncertainty. The computation of the $∆W_{j}$ vectors can be, again, formulated as a convex optimization problem having the same form achieved in the identification of the conventional IMs in (6). Therefore, the resulting optimization problem is:

(11) $$\begin{array}{l} \underset{∆W_{1},∆W_{2},\;\ldots ,\;∆W_{m}}{\min}J\;subject\;to\\ \;+y(k)-f_{nn}(\varphi (k),W_{nn})-\sum_{j=1}^{m}\left[|\varphi^{T}(k)|∆W_{j}\right]\beta_{j}\left(x_{p}(k)\right)<0\;\;\\ \;-y(k)+f_{nn}(\varphi (k),W_{nn})-\sum_{j=1}^{m}\left[|\varphi^{T}(k)|∆W_{j}\right]\beta_{j}\left(x_{p}(k)\right)<0 \end{array}\;$$

where, in this case, the optimization variables are the m×p parameters ($∆w_{ij},i=1,\ldots ,p,j=1,\ldots ,m$) in $∆W_{j}$, *i* = 1, …, *m*. Problem (11) can be set as a convex optimization problem in m×p parameters and solved using the same optimization tools used for the conventional IM in (6). The solution of the optimization problem (11) provides the *m* minimum uncertainty boxes components $∆w_{ij}$ that satisfy the consistency requirement. It should be emphasized that, as in the previous case, the LMI problem (11) is always solvable.

## 4. The Semiautonomous P92 Tecnam Aircraft and the Flight Data

**Figure 1:**
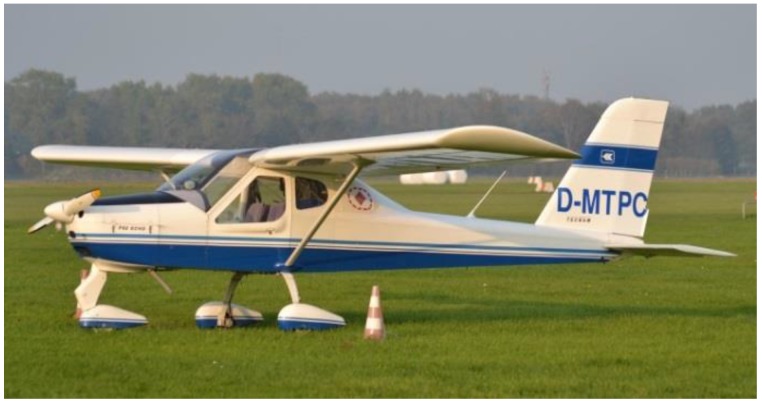
The semiautonomous P92 Tecnam aircraft.

Multiple flight data sets from the semiautonomous P92 Tecnam aircraft (shown in Figure 1) were used in this effort. The P92 is a single engine, high wing, general aviation light aircraft [13]. The aircraft mass is approx. 600 kg (1324 lb); the aircraft propulsion is provided by a 74 kw (98 hp) Rotax 912 ULS with a two blade fix pitch propeller allowing a maximum cruise speed of 219 km/h (118 kts) and an operational ceiling of 4267 m (14,000 ft). In the flight experiments, the aircraft was taken off and landed by the pilot while at altitude was remotely piloted from a ground station. Data from nine flights tests (labeled as flt-1, …, flt-9) were used.

**Figure 2:**
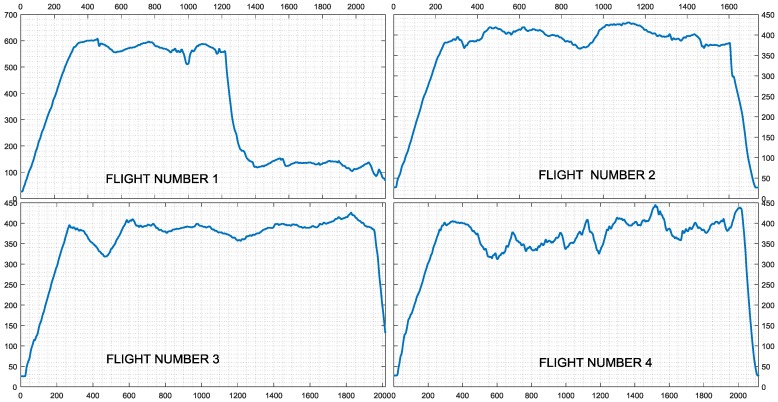
Altitude profiles of the four validation flights.

To evaluate the properties of the proposed FD schemes in every single phase of the flight, complete data records were considered, including take-off, initial climb, level flight, descent, approach, and landing. Each flight provides approximately 35 min of flight data. The data are all synchronized with a sampling period *T_s_* = 0.1 s Data from flt-#1 to flt-#5 (overall approx. 2.5 h) were used for model identification purposes and for the tuning of the fault detection systems, while the remaining four flights from flt-#6 to flt-#9 (overall approx. 2 h) were used for validation purposes. The altitude profiles of the aircraft for the four validation flights are shown in Figure 2.

It is observed that the validation flights contain a limited number of steady-state segments and a large number of maneuvers, essentially dynamic transients, such that the triggering of possible false alarms is more likely.

## 5. Modeling of the IMU Sensors

The design of FD schemes for the three IMU sensors of roll rate *p*(*k*), pitch rate *q*(*k*) and yaw rate *r*(*k*) is here considered. These signals were estimated as functions of other on-board available measurements. The initial selection of modeling inputs was performed simply selecting the signals that characterize the aircraft kinematic and dynamic response. The initial set of measurements consists of 13 signals $X_{0}(k)\in R^{13}$:

(12) $$X_{0}(k)=[\varphi (k),\theta (k),N_{x}(k),N_{y}(k),N_{z}(k),\alpha (k),\beta (k),V_{tas}(k),h(k),\delta_{e}(k),\delta_{a}(k),\delta_{r}(k),T_{Trotthe}(k)]\;$$

where φ(k),θ(k) are aircraft roll and pitch angles, $N_{x}(k),N_{y}(k),N_{z}(k)$ are the axial load factors, α(k),β(k) are the angle of attack and the sideslip angle, $\delta_{e}(k),\delta_{a}(k),\delta_{r}(k)$ are the deflections of elevator, aileron, and rudder, $T_{Trotthe}(k)$ is the throttle, h(k) is altitude, and $V_{TAS}(k)$ is the true airspeed. Considering possible dependency on the inverse of some regressors in *X*_0_(*k*) and the potential nonlinear dynamic characteristics of the system, the set of potential regressors was augmented with inverse and quadratic terms of the signals *X*_0_(*k*) obtaining a vector $X(k)=[X_{0}(k),1/X_{0}(k),X_{0}{\left(k\right)}^{2}]\in R^{39}$. Note that the nonlinear regressors were limited to quadratic terms to minimize the dimensionality of the overall regressor vector. Additionally, to evaluate the possible dependence of the model also on the delayed samples of $X_{0}(k)$ the set of regressors was augmented to include delayed values $x_{i}(k-m)$, i=1,…,13, with *m* = 1 s and *m* = 2 s, so that the resulting vector $X_{tot}(k)\in R^{65}$ provides 65 potential regressors, leading to:

(13) $$X_{tot}=[X_{1},\ldots ,X_{13},1/X_{1},\ldots ,1/X_{13},{X^{2}}_{1},\ldots ,{X^{2}}_{13},X_{1}(k-1),\ldots ,X_{13}(k-1),X_{1}(k-2),\ldots ,X_{13}(k-2)]$$

Finally, to limit the number of constraints in view of the solution of the optimization problems (6) and (11), the training data were down sampled (only in the IM model identification phase) to a sampling rate *T_s_* = 1 s.

It should be emphasized that the signals *p*(*k*), *q*(*k*) and *r*(*k*) were not included in *X*_0_(*k*) because, once again, the goal of the study was to generate prediction models that do *not* depend on the three potentially faulty signals. The advantage, as it will be clear in the results shown later, is that the residuals will not suffer of the previously described fault following effect and that, consequently, faults occurring on one (or more) sensor(s) are immediately detectable and identifiable. Therefore, with the proposed approach fault isolation is not an issue of concern.

### Regressors Selection via the Stepwise Method

This section focuses on the selection of suitable subsets of signals from the $X_{tot}(k)$ potential regressors set that are critical for the estimation of the prediction models for the roll rate $\hat{p}(k)$, the pitch rate $\hat{q}(k)$, and the yaw rate $\hat{r}(k)$. This goal was achieved using the ‘stepwise selection method’ described next. Stepwise regression is an iterative semiautomated data-driven method for developing a linear input-output model by successively adding or removing variables based on their statistical significance in a regression [18]. Starting from scratch, the method compares the explanatory power of incrementally larger and smaller models. At each step, the *p*-value of a *F*-statistic is computed to test the performance of the model with and without a potential term. The rational of the algorithm is to build an incremental complexity model including and maintaining in the model only the regressors having a probability less than a specified threshold (*p*-value) of having zero coefficients in the regression model. The threshold of the *p*-value has a direct impact on the selection process of the regressors; therefore, the value of the *p*-value was used as a tradeoff between model accuracy and model complexity. Specifically, the stepwise selection was performed collecting together data taken from 5 independent flights using a specific function of MATLAB. The selection of the most appropriate subset of regressors was based on the evaluation of the Root Mean Square Error (RMSE) index (where $RMSE=\sqrt{E({\left[y(k)-\hat{y}(k)\right]}^{2})}$) provided by the NN prediction models either in training and validation phase. The achieved training and validation results are shown in Table 1. It can be observed that the value of RMSE decreases by increasing the number of regressor in case of the training flight data. However, this is not true for the validation flights where indeed the RMSE increases for models featuring a large number of regressors. This clearly shows that very complex models suffer from over fitting problems and, for this reason, they should not be selected. Therefore, for all the models, the set of regressors which produced the smallest value of the RMSE index for the validation flight data was selected. Based on the results reported in Table 1 the final subset of regressor for the three sensor models is given by:

(14) $$\begin{array}{l} \varphi_{p}(k)=[1,\varphi (k),\theta (k),NN_{y}(k),\beta (k),\delta_{A},\varphi (k-1),\beta (k-1),\ldots ,\\ \delta_{A}(k-1),\varphi (k-2),NN_{y}(k-2),\delta_{A}(k-2),\delta_{R}(k-2)] \end{array}$$

(15) $$\begin{array}{l} \varphi_{q}(k)=[1,\varphi (k),\theta (k),NN_{z}(k),1/\alpha (k),1/V_{tas}(k),\ldots ,\\ \varphi^{2}(k),{V_{tas}}^{2}(k),\theta (k-1),NN_{z}(k-1),\theta (k-2),\beta (k-2)] \end{array}\;$$

(16) $$\begin{array}{l} \varphi_{r}(k)=[1,\varphi (k),NN_{y}(k),\beta (k),1/NN_{y}(k),\varphi^{2}(k),\ldots ,\\ N{N_{y}}^{2}(k),\beta^{2}(k),{T_{Thr}}^{2}(k),\beta (k-1),\varphi (k-2),\beta (k-2)] \end{array}\;$$

In the sets of regressors (14)–(16) the constant regressor “1” was also included to capture possible constant offsets in the models and/or to consider the effects of a bounded amplitude noise.

## 6. Identification of Nonlinear Regression Model

This section describes the procedure followed for the identification of the model (1) based on the regressors signals in (14)–(16) and the training data flights. The following NR models were thus identified

(17) $$\begin{array}{l} {\hat{p}}_{nn}(k)=f_{p}(\varphi_{p}(k),W_{nn\_p})\\ {\hat{q}}_{nn}(k)=f_{q}(\varphi_{q}(k),W_{nn\_q})\\ {\hat{r}}_{nn}(k)=f_{r}(\varphi_{r}(k),W_{nn\_r}) \end{array}\;$$

The above NR functions were approximated using a NN architecture [16]. Specifically, a two-layer Perceptron NN with five sigmoidal neurons in the first layer and one linear neuron in the output layer was used for each model. The number of neurons in the hidden layer was empirically selected after some empirical tests with the goal of avoiding overfitting problems while still having reliable estimates. A NN with five neurons in the hidden layer was set to be a good tradeoff for all three models. The feedforward NN was trained using the basic functions of the MATLAB NN toolbox. As for the LR models the *W_LRi_* coefficients were identified using simple LS estimation. The corresponding model identification performance measured in terms of the statistics of prediction error in the validation phase, are shown in Table 2.

It is observed that the LR models perform worse than the NR models. This is not surprising since the identification training data covers the entire flight including take-off, climbing, level flight, descent, approach, and landing. Therefore, it is reasonable to expect that a single linear model is not able to approximate accurately the aircraft response in all the different flight conditions while a nonlinear NN model can better cope with the nonlinearities of the dynamic response. For this reason, the analysis will be performed considering only the NR models in the following.

## 7. Identification of the Interval Models

This section describes the identification process for the conventional IMs and the FIMs that have been defined in Section 3.

### 7.1. Conventional Nonlinear Interval Models

Considering the conventional IMs, the upper and lower prediction interval bounds for the estimation for the three sensors can be readily computed from (3) resulting in:

(18) $$\begin{array}{ll} \bar{p}(k)=f_{p}(\varphi_{p}(k),W_{nn\_p})+|\varphi_{p}(k)|∆W_{NN-p} & \underline{p}(k)=f_{p}(\varphi_{p}(k),W_{nn\_p})-|\varphi_{p}(k)|∆W_{NN-p}\\ \bar{q}(k)=f_{q}(\varphi_{q}(k),W_{nn\_q})+|\varphi_{q}(k)∆|W_{NN-q} & \underline{q}(k)=f_{q}(\varphi_{q}(k),W_{nn\_q})-|\varphi_{q}(k)|∆W_{NN-q}\\ \bar{r}(k)=f_{r}(\varphi_{r}(k),W_{nn\_r})+|\varphi_{r}(k)|∆W_{NN-r} & \underline{r}(k)=f_{r}(\varphi_{r}(k),W_{nn\_r})-|\varphi_{r}(k)|∆W_{NN-r} \end{array}$$

Specifically in (18) the previously identified NR models (17) were used as central models while the interval uncertainty was computed solving the optimization problem (6) for the 3 sensors. These problems can be efficiently solved using a number of optimization software tools. In our study we used the functions of the MATLAB Linear Matrix Inequalities (LMI) toolbox [22]. The optimization variables $\left[∆{\bar{w}}_{1},∆{\bar{w}}_{2},\ldots ,∆{\bar{w}}_{p}\right]$ defining the uncertainty parameter box were derived by solving (for each one of the 3 models in (17)) the convex optimization problem (6) using the cost function *J* previously defined in Section 3.2. Table 3 shows the values of the uncertainty intervals computed for each regressor of the three models resulting from the optimization.

Considering the computational load required for the solution of the LMI problems in (6) with 2N = 21,620 constraints, the problem was solved using a Core-i7 Intel processor with 8 Giga RAM in less than a minute for each of the three IMU sensors. This clearly shows that the proposed technique can be effectively implemented with batch of data of average size. The possibility of applying the proposed technique to larger optimization problem strictly depends on the availability of computational power and on the efficiency of the solving algorithm. Large scale and distributed optimization is a very active and quickly evolving field of research. Interested readers are referred to [23,24].

### 7.2. Identification of the Fuzzy Interval Models

**Figure 3:**
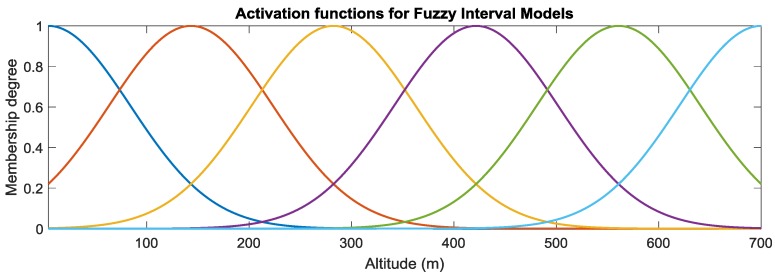
Gaussian membership functions associated to the FIM depending on the flight height h.

The FIMs were defined as function of the aircraft altitude *h*. In this context Gaussian membership functions (GMF) were used for the characterization of the fuzzy membership functions. These functions are thus parametrized as function of the altitude *h* (that is *x_p_ = h*) resulting in:

(19) $$\mu_{A_{j}}\left(x_{p}\right)=\exp(-\frac{{\left(c_{j}-x_{p}\right)}^{2}}{2\sigma_{j}^{2}})j=1,\ldots ,m$$

where $\mu_{A_{j}}(x_{p}):R\to \left[0,1\right]$ represents the activation of the *i*-th fuzzy membership function at altitude $x_{p}$; while $c_{j}$ and $\sigma_{j}^{}$ are the centre and width of the *j*-th Gaussian fuzzy set $A_{j}$, respectively. The aircraft operative range is below 700 m; therefore, the GMFs were defined in the range [0, 700] m. Specifically, the *m* = 6 GMFs shown in Figure 3 were implemented.

Using the above GMFs the LMI problem (11) was solved using the same software used for the conventional IM approach. For each sensor, the results of the optimization produced a *p* × 6 matrix of coefficient’s whose rows characterize the six local FIM models. Similarity to (18) the FIMs provide the following upper and lower bounds for the three sensors:

(20) $$\begin{array}{ll} \bar{p}(k)=f_{p}(\varphi_{p}(k),W_{nn\_p})+|\varphi_{p}(k{)}^{T}|∆W_{NN-p}\beta (x_{p}(k)) & \underline{p}(k)=f_{p}(\varphi_{p}(k),W_{nn\_p})-|\varphi_{p}(k{)}^{T}|∆W_{NN-p}\beta (x_{p}(k))\\ \bar{q}(k)=f_{q}(\varphi_{q}(k),W_{nn\_q})+|\varphi_{q}(k{)}^{T}|∆W_{NN-q}\beta (x_{p}(k)) & \underline{q}(k)=f_{q}(\varphi_{q}(k),W_{nn\_q})-|\varphi_{q}(k{)}^{T}|∆W_{NN-q}\beta (x_{p}(k))\\ \bar{r}(k)=f_{r}(\varphi_{r}(k),W_{nn\_r})+|\varphi_{r}(k){|}^{T}∆W_{NN-r}\beta (x_{p}(k)) & \underline{r}(k)=f_{r}(\varphi_{r}(k),W_{nn\_r})-|\varphi_{r}(k{)}^{T}|∆W_{NN-r}\beta (x_{p}(k)) \end{array}$$

where the $∆W_{NN-p},∆W_{NN-q},∆W_{NN-r}$ are suitable dimension matrices (p×6) containing the optimized model parameters and $\beta (x_{p})$ is a 6×1 vector containing the activation of the *m* fuzzy rules at height *h = x_p_*. As expected the FIMs are more flexible in capturing the uncertainty at the different flight altitude thanks to the fuzzy fusion of the 6 local linear modes. This was confirmed in Table 4 where the values of cost function *J* for the four validation flights are reported. Table 4 shows indeed a reduction of *J* of approx. [40–50%] for all the angular velocities when compared with the conventional IM performance.

**Figure 4:**
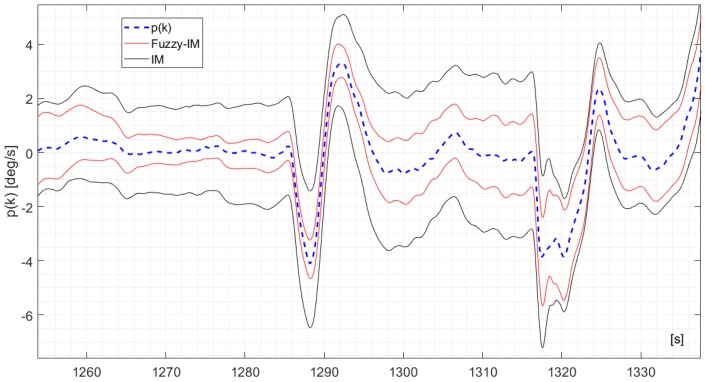
The measured *p*(*k*) signal and the time varying upper bounds $\bar{p}(k)$ and lower bounds $\underline{p}(k)$ derived for the IM applying Equation (18) and for the FIM applying Equation (20).

**Figure 5:**
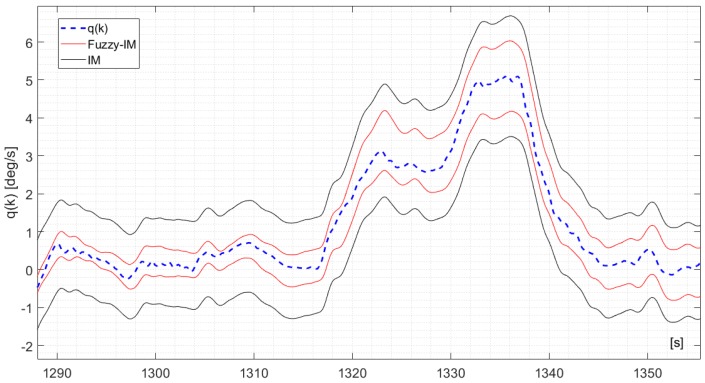
The measured *q*(*k*) signal and the time varying upper bounds $\bar{q}(k)$ and lower bounds $\underline{q}(k)$ derived for the IM applying Equation (18) and for the FIM applying Equation (20).

**Figure 6:**
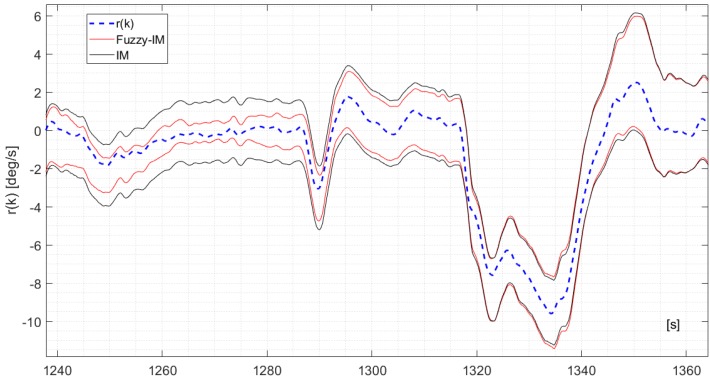
The measured *r*(*k*) signal and the time varying upper bounds $\bar{r}(k)$ and lower bounds $\underline{r}(k)$ derived for the IM applying Equation (18) and for the FIM applying Equation (20).

To further show the benefits of the FIMs compared to the IMs, Figure 4 shows a 70 s. segment of the measured *p*(*k*) signal derived from validation flight #1 in fault-free conditions and the corresponding upper and lower bounds $\bar{y}(k)$ and $\underline{y}(k)$ computed using (3) and (10) for the IM and FIM approaches. It is clear that the FIM provides a tighter approximation of the uncertainty bounds than the IM; additionally no false alarms are reported. A similar trend was observed also for the *q*(*k*) and the *r*(*k*) sensors with results shown in Figure 5 and Figure 6 respectively (considering different time segments of the same validation flight #1). It should be pointed out that the difference in performance between the FIM and the IM models are less noticeable for the *r*(*k*) signal.

### 7.3. Residual Computation for the Interval Models

The fault detection logic used in this study was inspired by the approach proposed in [11]. Specifically, the residual signal is defined as the difference between the measurement y(k) and the central nominal model, which is the nonlinear models defined in (17):

(21) $$r(k)=y(k)-f_{nn}(\varphi (k),W_{nn})\;$$

Considering now the lower and upper bounds for the IM and FIM defined in (18) and (20), the upper and lower bounds for the residuals can be derived as follows:

(22) $$\begin{array}{l} \bar{r}(k)=\bar{y}(k)-f_{nn}(\varphi (k),W_{nn})\\ \underline{r}(k)=\underline{y}(k)-f_{nn}(\varphi (k),W_{nn}) \end{array}\;$$

The $\bar{r}(k)$ and $\underline{r}(k)$ signals provide *time varying* bounds for the residual signal that depend on the value of the current regressors in φ(k) and on the IM and FIM uncertainty. The fault detection test simply consists in evaluating the condition

(23) $$r(k)\in [\underline{r}(k)\bar{r}(k)]\;$$

The event that condition (23) is not satisfied implies that the current measurement is not ‘explained’ by the IM or FIM and, therefore, a failure detection is declared.

### 7.4. Fixed Thresholds Computation

A comparative study was conducted considering a fault detection method based on the standard central models (17) with the goal of evaluating the performance of the fault detection schemes based on the proposed IM and FIM approaches. In this case the residual *r*(*k*) is compared against a fixed detection threshold derived following a statistical analysis of the residuals itself based on experimental fault free data. From the analysis of the residual *r*(*k*) signals it was seen that they do not exhibit a white spectrum; in fact, in general, they are characterized by a significant autocorrelation caused, mainly, by the modeling uncertainties. On the other hand, the condition of uncorrelated residual is one of the requirements for most fixed threshold statistical change detectors [25]. Therefore, based on the training data, a residual whitening filter was designed to remove correlation from the raw residual. The withering filter is based on the design of a one-step-ahead predictor that is defined as follows:

(24) $$r(k)=\hat{r}(k)+\varepsilon_{r}(k)=\sum_{i=1}^{N_{w}}d_{i}\cdot r(k-i)+\varepsilon_{r}(k)\;$$

In (24) the residual *r*(*k*) has been modeled as an AUTO REGRESSIVE (AR) process of order *N_w_* where $d_{i}$ are the AR parameters and $\varepsilon_{r}(k)$ is a white noise. The term $\hat{r}(k)$ can be interpreted as a one-step-ahead prediction of *r*(*k*), and $\varepsilon_{r}(k)$ as the prediction error. The $d_{i}$ coefficients were estimated applying the LS method to the experimental prediction error of the training data. Once the $d_{i}$ coefficients have been computed, the prediction error sequence $\varepsilon_{r}(k)$ derives immediately from (24) as $\varepsilon_{r}(k)=r(k)-\hat{r}(k)$ and can be computed as the output of the linear filter $\varepsilon_{r}(z)=W_{FIR}(z)r(z)$ where $W_{FIR}(z)=1-d_{1}z^{-1}-d_{2}z^{-2},\ldots ,-d_{N}z^{-N}$ and *z*^−1^ is the discrete time shift operator. If the modeling assumptions in (24) are valid, then $\varepsilon_{r}(k)$ is a white noise sequence and *W_FIR_*(*z*) is a whitening filter for *r*(*k*). The $\varepsilon_{r}(k)$ signal will be named, hereafter, as the ‘whitened residual’ *r_w_*(*k*).

The length *N_w_* of the filter *W_FIR_*(*z*) was determined by computing the filtered residual *r_w_*(*k*) for increasing values of *N_w_* and applying the Ljung–Box test [26] to evaluate whether *r_w_*(*k*) does not exhibit significant autocorrelation with significance level of at least 0.05. For the 3 considered sensors the values *N_w_* = 25 was found to satisfy the Ljung–Box test; therefore, *N_w_* = 25 was used in the experiments for all the residuals.

The fixed fault detection thresholds named also as UCL (upper control limit) and LCL (lower control limit) were derived from the Experimental Cumulative Distribution Function (ECDF) of the whitened residual signal *r_w_*(*k*) as function of the desired false alarm probability *P_FA_* applying the procedure proposed in [27], where *P_FA_* is the probability that |*r_w_*(*k*)| exceeds the detection threshold at fault free conditions. Considering the fact that for the training data the IM and FIM approaches are able to guarantee zero false alarms in fault free conditions, in order to have a fair comparison we have considered a very low probability of false alarms equal to 0.0001 to compute the FD threshold for the signal *r_w_*(*k*). Table 5 reports, for the three sensors, the experimental UCL and LCL that guarantee the defined *P_FA_*. The approach here proposed will be referred to as the fixed thresholds model (FTM) in the next sections.

## 8. Fault Modelling

Failures affecting sensors are typically classified on the basis of their effects on the system output signals [28]. Additive failures are usually modelled as independent exogenous signals acting on the plant outputs that are zero at fault free conditions and different from zero when a fault occurs. Multiplicative faults instead are usually associated with the variation of the magnitude of some internal physical parameters, components, or known inputs of the system. Sensor faults are very often modelled as additive failures [29]. Specifically the failures on the IMU were modelled as additive signals *f*(*k*) occurring at a generic time step *k_f_*. The resulting faulty signal is given by:

(25) $$y_{faulty}(k)=y(k)+f(k-k_{f})\;$$

To evaluate hard, soft (incipient), and limited duration sensor failures, the fault *f*(*k*) was modelled as the response of a first order low-pass filter with time constant τ to a rectangular input $rect(t,T_{rect})$

(26) $$f(t)=\frac{∆f}{1+s\tau }\;rect(t,T_{rect})\;$$

where Δ*f* is the steady state fault amplitude and *T_rect_* is the duration of the rectangular input. By varying the parameter is possible to simulate abrupt and slowly developing failures while through changes in *T_rect_* it is possible to vary the failure duration.

## 9. Analysis and Validation of the Fault Detection Schemes Using Real Flight Data

Fault detection tests were performed on the three IMU sensors using the four validation flights. Once again, the data from the validation flights were not used for training purposes. For each flight ‘artificial’ additive hard “rectangular shaped” failures (26) were injected for a temporal interval (*T_rect_*) equal to the 10% of the entire flight duration between the 65% and the 75% time mark of the flight, implying that the temporary failure occurs at level flight conditions. Before the 65% and after the 75% time mark no failures occurred. The rectangular fault shape allows evaluating, in the same flight, the robustness to false alarms (the fault is not active for 90% of the flight) as long as the fault detectability during the 10% period when the rectangular fault is active. For the three IMU sensors the following performance indices were evaluated to quantify the effectiveness and the robustness of the FD schemes:

- **Percent False Alarms index** (%FA), that is the % of time the residual signal is above the FD thresholds in the time interval [0–65%] and [75–100%] when the fault is zero.
- **Percent True Detections index** (%TD), that is the % of time the residual signal is above the FD threshold in the time interval [65–75%] when the fault is active.
- **First Detection Delay** (FD_Delay_), that is the time the residual takes to exceed the FD thresholds for the first time starting from the fault initial instant at the 65% time mark; if the residual never falls outside the intervals during the period of fault this value is conventionally set equal to the duration of the failure. In the following analysis the %TD and the FD_Delay_ have been also indicated as “true positive” (%) and “first detection” (s) respectively.

### 9.1. IMU Sensors Fault Detection Results

**Figure 7:**
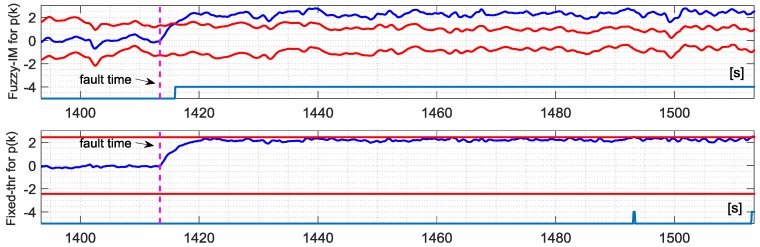
Evolution of the NR residual for the *p*(*k*) sensor and the corresponding FD intervals as long as the binary fault indicator for the FIM approach (**Upper**) and fixed-thresholds approach (**Lower**) in case of validation flight number 1.

**Figure 8:**
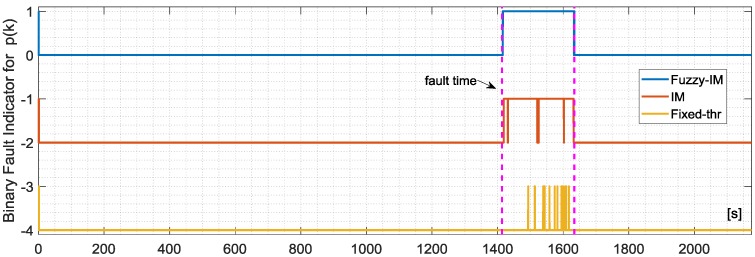
Evolution of the FIM, IM and FTM binary fault indicators for validation flight 1 in case of hard failure on the *p*(*k*) sensor of amplitude 2.29 deg/s active in a time window of about 3 min indicated by the vertical dashed lines.

The first validation tests were performed to evaluate the fault sensitivity and the robustness to false alarms of the *p*(*k*) sensor. Hard failures (assuming a very small time constant τ = 0.01 s in (26)) were added in the time interval [65–75%] considering fault amplitudes Δ*p* in the range from 0 to 4 deg/s with increments of 0.1 deg/s. Figure 7 shows a comparison between the FIM and the FTM approaches defined in paragraphs 7.2 and 7.4 respectively, in case of a fault with amplitude 2.3 deg/s in a time window of approx. 3 min. It is observed that the FIM approach is more effective than the FTM. In fact, for this fault amplitude the FTM scheme is not able to detect the failure unless for a very short period after 75 s while the FIM method provides a prompt and persistent detection of the failure. Figure 8 shows a comparison of the evolution of the binary fault indicator signal (a signal set to 1 when the residual exceeds the thresholds and 0 otherwise) during the entire validation flight #1 for the 3 methods FIM, IM and FTM, in case of additive failure of amplitude Δ*f* = +2.3 deg/s. The analysis of the figure clearly shows that FIM provides better FD performance in terms of true positive and first detection delay. The percentage of false alarms is less than 0.4% for the all the three methods. It is also observed that in the temporal interval when the fault is active (between the two vertical dashed lines in the Figure 8) the FIM scheme provides a more persistent detection compared to the other methods. It can be concluded that, in this case, the FIM method, based on fuzzy time varying detection bounds, is more effective than the conventional IM and FTM approaches. The statistical results achieved in the four validation flights are reported in Table 6 where the superiority of the FIM methods is confirmed in all the four validation flight. In fact for the FIM the mean percentage of true positive is 99.05% while for IM and FTM it is equal to the 73.35% and 11.86% respectively. Additionally, the performance of the FIM are also superior in terms of the mean detection delay with less than 2 s for the FIM, 3.85 s for the conventional IM, and 20.83 s for the FTM.

**Figure 9:**
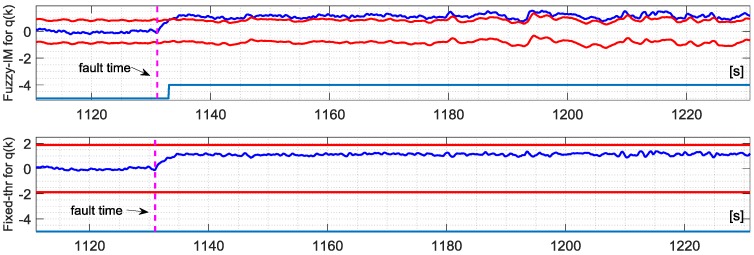
Evolution of the NR residual for the *q*(*k*) sensor and the corresponding FD intervals as long as the binary fault indicator for the FIM approach (**Upper**) and Fixed-thresholds approach (**Lower**) in case of validation flight number-1.

The analysis of the FD performance for the *q*(*k*) sensor in the presence of hard failures was performed using the same approach applied for the *p*(*k*) sensor using, again, the four validation flights. The achieved results essentially confirm the previous results for the *p*(*k*) sensor. Specifically Figure 9 shows the FD detection performance for the FIM and FTM approaches in the presence of an additive hard failure of 1.1 deg/s on the *q*(*k*) where it can be observed that the FIM is able to provide a prompt and persistent detection while the FTM misses completely the same failure.

**Figure 10:**
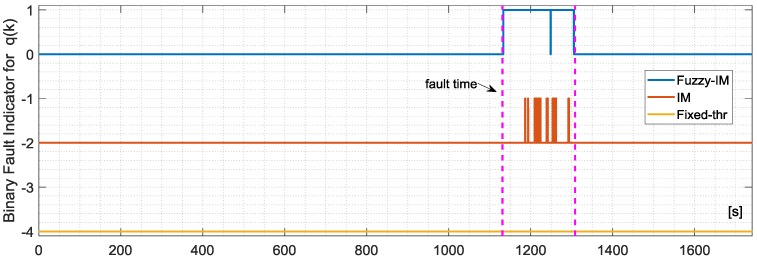
Evolution of the FIM, IM and FTM BINARY FAULT indicators for validation flight-1 in case of hard failure on the *q*(*k*) sensor of amplitude 1.15 deg/s active in a time window of about 3 min indicated by the vertical dashed lines.

The binary fault signature for the three methods are shown in Figure 10. The statistical results are reported in Table 7; a detailed analysis of the results reveal that it is apparent that the Fuzzy method still provides a remarkable improvement with respect the other methods, in particular compared to the FTM which was not able to detect small amplitude faults of 1.1 deg/s in all the validation flights. For the FIM the percentage of true positive is 89.20% with mean first detection delay of 2.45 s while for conventional IM the delay results of 14.18 s while the true positive are 13.33%.

**Figure 11:**
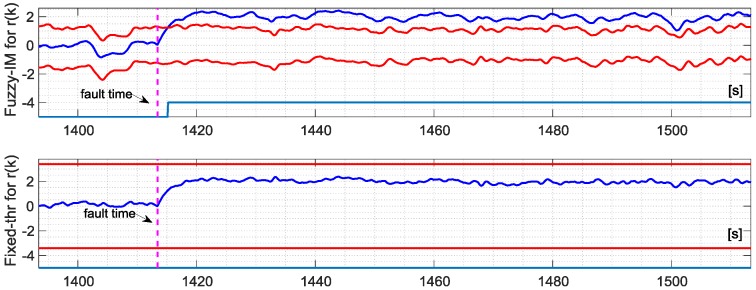
Evolution of the NR residual for the *r*(*k*) sensor and the corresponding FD intervals as long as the binary fault indicator for the FIM approach (**Upper**) and fixed-thresholds approach (**Lower**) in case of validation flight number 1.

**Figure 12:**
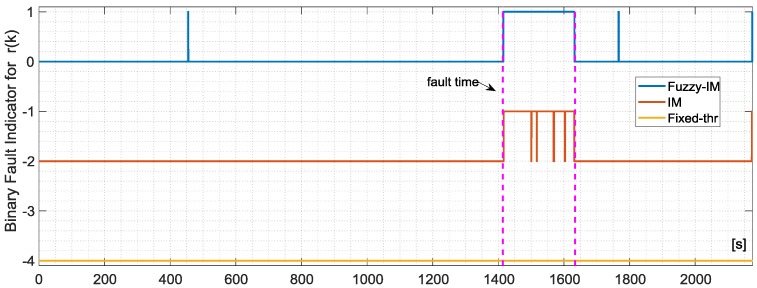
Evolution of the FIM, IM and FTM binary fault indicators for validation flight 1 in case of hard failure on the *r*(*k*) sensor of amplitude 2.005 deg/s active in a time window of about 3 min indicated by the vertical dashed lines.

The same analysis has been performed also for the sensor *r*(*k*) in case of fault amplitude of 2.0 deg/s. Figure 11 and Figure 12 still confirms that FIM is more sensitive than the FTM; in fact, this last method is not able to the detect the failure on the *r*(*k*) sensor at all. Table 8 confirms the superiority of the FIM approach in particular with respect to FTM method. On the other side, the performance comparison between FIM and conventional IM did not reveal a significant improvement; in fact, for the FIM the percentage of true detections is 84.8% along with a first detection delay of 1.825 s, while for the IM the percentage of true detections is 76.32% with a detection delay of 2.925 s. Indeed Table 4 highlights that, in the specific case of the *r*(*k*) sensor, the FIM produces a less evident reduction in the cost function *J* with respect to the *p*(*k*) and *q*(*k*) sensors when compared with the corresponding conventional IMs.

### 9.2. Fault Sensitivity Analysis

**Figure 13:**
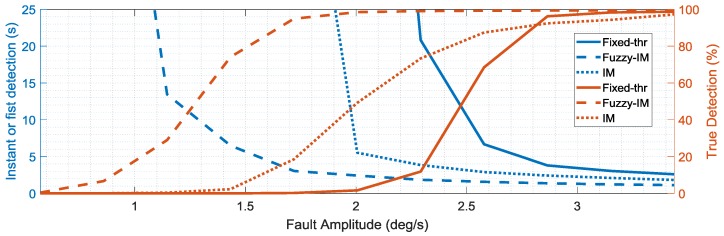
FD performance indexes for the *p*(*k*) sensor as function of the fault amplitude (mean values evaluated for the four validation flights).

**Figure 14:**
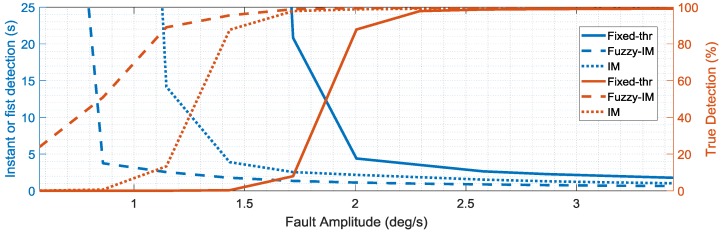
FD performance indexes for the *q*(*k*) sensor as function of the fault amplitude (mean values evaluated for the four validation flights).

**Figure 15:**
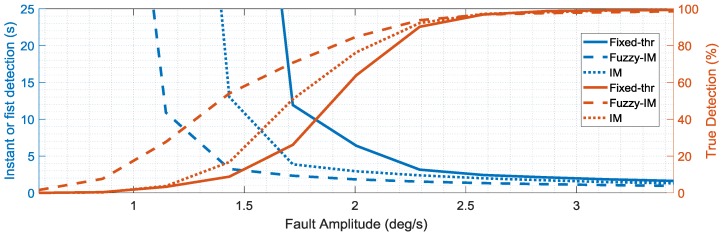
FD performance indexes for the *r*(*k*) sensor as function of the fault amplitude (mean values evaluated for the four validation flights).

To assess the sensitivity of the different schemes, the percent true detections index and the fault detection delay indices were evaluated systematically for increasing fault amplitudes. The mean values of these indexes (obtained from the four validation flights) are reported in the Figure 13, Figure 14 and Figure 15 as functions of the fault amplitudes for the three sensors. In terms of the *p*(*k*) sensor, Figure 13 clearly highlights the impact of the fault amplitude on the failure detection delay and on the persistency of the failure detection when the fault is active. Clearly, the fault amplitude is higher, the failure detection is quicker, and the percent true detection index is higher for all the methods. It is apparent that the FIM approach performs significantly better than conventional IM and FTM approaches in terms of both smaller detection delays and percentage of true detections. The analysis of the results in Figure 13 also reveals that the FIM approach allows a significant improvement of the performance in terms of shorter detection delay and higher percent true detections especially in the case of small amplitude faults with amplitude less than 2 deg/s. In fact, the IM approach starts detecting failures with an acceptable level of % true detection only for fault amplitudes larger than 2.5 deg/s, while, for the FTM approach the detection occurs only for fault amplitudes larger than 3 deg/s. Figure 14 and Figure 15 confirm similar trends for the FD sensitivity indexes for the *q*(*k*) and *r*(*k*) sensors.

To complete the analysis. an additional study was performed by evaluating, throughout the entire flight, the % of the false alarms produced during the four validation flights. This study allows to clarify whether the improvement in the fault sensitivity by the FIM is achieved at the expense of a deterioration of performance in terms of % false positive detections. The comparative results for the FIM, IM. and FTM approaches are reported in Table 9. The analysis of the results reveals that the three methods provide similar % level of false alarms. This last consideration is particularly relevant since it proves that the increase of the FD sensitivity of the FIM approach is not achieved at the expense of an increase of the false alarm rate.

### 9.3. Comparison with Other State-of-the-Art Techniques

To evaluate the performance of the proposed robust data-driven methods it is also important to perform comparison with other more conventional state-of-the-art FD methods that rely on physical models. This study was recently performed in [13] by the authors for the true airspeed (TAS) sensor. In that study, two extended Kalman filter (EKF) approaches were evaluated to estimate the TAS signal within a FD scheme, showing similar performance (compared to the proposed approaches) only in the presence of large amplitude step failures. On the other side the performance of the EKFs methods deteriorates drastically in case of incipient failures. A possible motivation is that the feedback contribution in the correction term of the EKFs introduces a typical fault-following effect that has the effect of ‘desensitizing’ the residual and, thus, making difficult to detect small amplitude failures. Although extensive and time consuming texts were performed to improve the fault sensitivity, a conclusion of the effort described in [13] was that it was not possible to define EKFs settings that are satisfactory for all the validation flights. The lack of robustness of the EKF methods probably originates from the fact that the signals used in EKFs are affected by significant noise, sensor dynamics, sensor offset, and time delays that concur in the generation of extremely noisy and correlated residuals even in fault free conditions. All these practical drawbacks, at least for our validation data, have a direct and negative effect on the FD performance of the considered model-based methods. Therefore, because of the trends previously described, a similar comparative study was not repeated in the present study for the considered IMU sensors.

## 10. Conclusions

The purpose of this study was to propose the design of a data-driven scheme for the robust detection of failures of IMU Sensors. To cope with the significant modeling uncertainties associated with the aircraft flying at different points of a flight envelope a robust FD scheme based on FIMs has been proposed. The goal is to improve the performance in terms of detection delay and percentage of true alarms with respect to a conventional IM approach. Specifically, nonlinear in the parameters neural network models were developed to capture the nominal response of the IMUs while an additive linear in the parameters FIM model was used to capture the uncertainty as function of the aircraft altitude. The FIMs and IMs identification was performed following the principle of *consistency* with all the experimental data and the principle of *tightness* of the parametric intervals. The approach naturally leads to the formalization of a constrained LMI optimization problem that produced optimized experimental FIMs and IMs characterized by minimum mean prediction intervals. The data-driven philosophy has been also applied for the selection of the best regressors for the FIMs and IMs. The validation of the FD schemes was conducted using a large data set of actual flight data from a P92 Tecnam aircraft, where a detailed comparison with conventional IM and fixed thresholds FTM approaches was performed. The analysis of the validation results has clearly shown that the FD schemes based on FIMs are more effective than conventional IM and FTM methods in terms of missed detection, minimum detectable faults, and detection delays, while levels of false alarms are in the same order of magnitude for all the three methods for the considered IMU sensors. The proposed data-based approach can be applied without any loss of generality and without significant conceptual modifications to FD problems for a variety of different applications.

## Figures and Tables

**Table 1:** RMSE index evaluated with the validation and trading flight data and the corresponding number of regressors (*N_regr_*) selected by the stepwise algorithm for different *p*-value.

*p(k)*	*N-regr*	4	5	6	11	12	14	26
	*RMSE validation*	0.057	0.0181	0.0162	0.0143	**0.0132**	0.0146	0.245
	*RMSE train*	0.0485	0.0216	0.0198	0.0157	0.0152	0.0142	0.0128
***q*(*k*)**	*Nregr*	3	5	6	9	**11**	14	24
	*RMSE validation*	0.0318	0.0298	0.0291	0.0286	**0.0272**	0.0307	0.0342
	*RMSE train*	0.0328	0.023	0.0212	0.0185	0.0181	0.0177	0.0172
***r*(*k*)**	*N-regr*	3	5	6	9	**11**	13	20
	*RMSE validation*	0.0325	0.0294	0.0285	0.0272	**0.0261**	0.0266	0.0283
	*RMSE train*	0.0307	0.0293	0.0272	0.0253	0.0243	0.024	0.0217

**Table 2:** Performance for Linear and Nonlinear models in validation phase.

	p(k) [deg/s] $e(k)=p(k)-\hat{p}(k)$			q(k) [deg/s] $e(k)=q(k)-\hat{q}(k)$			r(k) [deg/s] $e(k)=r(k)-\hat{r}(k)$		
	Mean	Std	|Max|	Mean	Std	|Max|	Mean	Std	|Max|
LR	0.221	0.226	2.072	0.121	0.189	2.356	0.306	0.349	3.086
NR	0.185	0.202	1.953	0.099	0.116	1.941	0.237	0.246	3.025

**Table 3:** Amplitudes of the IM parameters for the three IMU sensors.

	∆*W_NN-p_*	∆*W_NN-q_*	∆*W_NN-r_*
$∆{\bar{w}}_{0}$	7.79 × 10^−2^	1.50 × 10^−10^	1.05 × 10^−1^
$∆{\bar{w}}_{1}$	1.06 × 10^−9^	1.51 × 10^−9^	8.85 × 10^−3^
$∆{\bar{w}}_{2}$	2.57 × 10^−10^	1.09 × 10^−9^	1.10 × 10^−9^
$∆{\bar{w}}_{3}$	6.79 × 10^−10^	2.10 × 10^−10^	2.16 × 10^−10^
$∆{\bar{w}}_{4}$	2.85 × 10^−1^	6.61 × 10^−10^	2.53 × 10^−3^
$∆{\bar{w}}_{5}$	9.76 × 10^−2^	1.10 × 10^0^	1.45 × 10^−9^
$∆{\bar{w}}_{6}$	8.29 × 10^−10^	7.18 × 10^−2^	1.40 × 10^−8^
$∆{\bar{w}}_{7}$	7.56 × 10^−10^	1.21 × 10^−10^	2.49 × 10^−1^
$∆{\bar{w}}_{8}$	6.51 × 10^−10^	3.55 × 10^−9^	1.53 × 10^−10^
$∆{\bar{w}}_{9}$	6.88 × 10^−10^	2.10 × 10^−10^	1.60 × 10^−10^
$∆{\bar{w}}_{10}$	9.77 × 10^−10^	8.44 × 10^−10^	3.78 × 10^−10^
$∆{\bar{w}}_{11}$	1.38 × 10^−9^	1.21 × 10^−9^	1.76 × 10^−10^
$∆{\bar{w}}_{12}$	3.38 × 10^−10^	-	-

**Table 4:** Mean value of the cost function J for the four validation flights in case of Conventional IM and FIM approaches.

	*p*(*k*) [deg/s]	*q*(*k*) [deg/s]	*r*(*k*) [deg/s]
	J	J	J
IM	0.404	0.255	0.331
FIM	0.246	0.115	0.214

**Table 5:** Fixed fault detection thresholds for the three IMU sensors.

*p*(*k*) [deg/s]		*q*(*k*) [deg/s]		*r*(*k*) [deg/s]	
LCL	UCL	LCL	UCL	LCL	UCL
−2.45	2.45	−1.88	1.88	−1.89	1.89

**Table 6:** Mean FD performance in the four validation flights for the sensor *p*(k).

	FIM Fault: 2.3 [deg/s]	IM Fault: 2.3 [deg/s]	FTM Fault: 2.3 [deg/s]
*True Positive* (*%*)	99.05	73.35	11.86
*First Detection* (*s*)	1.85	3.85	20.83

**Table 7:** Mean FD performance in the four validation flights for the sensor *q*(k).

	FIM Fault: 1.1 deg/s	IM Fault: 1.1 deg/s	FTM Fault: 1.1 deg/s
*True Positive (%)*	89.20	13.33	0
*First Detection (s)*	2.45	14.18	/

**Table 8:** Mean FD performance in the four validation flights for the sensor *r*(*k*).

	FIM Fault: 2.0 deg/s	IM Fault: 2.0 deg/s	FTM Fault: 2.0 deg/s
*True Positive* (*%*)	84.8	76.32	63.74
*First Detection* (*s*)	1.825	2.925	6.4

**Table 9:** Mean performance for the four validation flights in terms of % false alarms.

	*p*(*k*) [%] False Alarms	*q*(*k*) [%] False Alarms	*r*(*k*) [%] False Alarms
IM	0.145	0.158	0.161
FIM	0.151	0.161	0.172
FTM	0.141	0.142	0.173
